# The dynamics of coffee production in Brazil

**DOI:** 10.1371/journal.pone.0219742

**Published:** 2019-07-23

**Authors:** Bruno Volsi, Tiago Santos Telles, Carlos Eduardo Caldarelli, Marcia Regina Gabardo da Camara

**Affiliations:** 1 Department of Agronomy, Universidade Estadual de Londrina, Londrina, Paraná, Brazil; 2 Department of Economics, Instituto Agronômico do Paraná, Londrina, Paraná, Brazil; 3 Department of Economics, Universidade Estadual de Londrina, Londrina, Paraná, Brazil; Indiana State University, UNITED STATES

## Abstract

Coffee is a crop of significant importance for Brazilian agrobusiness. There is evidence that both the geographic distribution of coffee production, and the varieties of coffee produced, have changed throughout Brazil over the course of time. Furthermore, it appears that these developments are associated with structural changes resulting from reductions in government intervention and its effects on prices in the coffee market, which has established a new dynamic of coffee production in the country. In this context, this study’s objective is to analyze the dynamics of coffee production in Brazil, to identify the Brazilian micro-regions specializing in coffee activities, and to track how the spatial distribution of these micro-regions has varied over time. In so doing, the study aims to identify defining economic characteristics of primary coffee-producing regions. Drawing primarily on data from the Brazilian Institute of Geography and Statistics, the study proceeds by applying Pearson correlation, Granger causality test, location quotient, principal components, and clustering analyses to explore how, during the 1984–2015 period, significant changes occurred in the distribution of regions specializing in coffee production. States such as Paraná and São Paulo, historically important coffee producers, declined in importance, leaving only a few micro-regions in these states specialized in coffee production. During the 2014/15 biennium, 80% of the coffee-specialized micro-regions were concentrated in the states of Minas Gerais, Bahia, Rondônia, and Espírito Santo. Minas Gerais and Bahia primarily produced arabica coffee, while Rondônia specialized in conilon (robusta) coffee. Overall, coffee produced in Brazil improved in quality and value-added over this period.

## Introduction

Brazil is the largest producer and exporter of coffee in the world. According to data from the Municipal Agricultural Survey (PAM), published by the Brazilian Institute of Geography and Statistics (IBGE), approximately 50.3 million sixty-kilogram bags of coffee were collected in Brazil during the 2016 harvest, with 42.5 million of these bags containing arabica coffee and 7.8 million containing conilon (robusta) coffee [1]. Based on data from the Ministry of Industry, International Commerce, and Services, the revenue generated from coffee exports in 2016 totaled US$ 4.84 billion, and primary export destinations included Germany, the United States, Italy, and Japan.

Coffee cultivation has evolved in significant ways throughout the course of Brazil’s historical and economic development, especially in terms of location of production. The coffee cultivation in Brazil began in the Northern region–state of Pará–in the 18^th^ Century, and later shifted toward the states of Rio de Janeiro and São Paulo (along Vale do Paraíba) [2]. Around 1850, cultivation spread rapidly toward the Serra da Mantiqueira and Santos. Later, in the 20^th^ Century, coffee cultivation continued its expansion into the states of São Paulo and southern Minas Gerais, Espírito Santo, Paraná, and even into Brazil’s Northern region, in the state of Rondônia [3]. Throughout this expansionary period, the Brazilian economy as a whole was tightly coupled with the coffee economy, and the coffee market was highly regulated by the Brazilian federal government until the mid-1990s.

Between 1952 and 1989, the Brazilian Coffee Institute (IBC), an authority tied to the Ministry of Industry, International Commerce, and Services, was responsible for regulation, control, and strategic coordination along the coffee value chain, from production to domestic and international commercialization, including incentive policies that absorbed internal surpluses and guaranteed fixed retail prices for coffee beans [4]. The IBC functioned by acquiring and stockpiling coffee beans produced in Brazil with the aim of regulating supply and demand and moderating price fluctuations. The IBC supplied lower-grade coffee beans to roasters at subsidized prices for sale in the Brazilian domestic market, while guiding higher-quality beans toward export. The breakdown of the international coffee agreement in 1989 associated with the dissolution of the IBC in 1990 brought these regulatory policies to an end. With this deregulation, the Brazilian coffee sector has since been fully exposed to the free market and the coffee growers experienced a long period of crisis and low levels of prices.

With the end of large-scale government intervention in the Brazilian coffee market, the sector was forced to reinvent itself. Coffee production systems were modernized and began to adopt increasingly innovative production techniques in the race to improve competitivity through differentiation in product quality [5], cost reductions, and even the creation of internal management mechanisms within firms, all with the goal of achieving client satisfaction and confidence [6]. In addition, coffee producers began to produce in regions that were climatically more favorable to cultivation [7]. Furthermore, producers sought to insert themselves into specific consumer markets and to increase the value-added of their product by producing specialty coffees with seals of quality and geographical certifications [8].

While coffee has undoubtedly been one of the most important crops throughout the history of Brazil, it has recently declined in importance relative to other crops, primarily grains. Therefore, it is important to analyze the dynamics of Brazilian coffee production since 1990 in order to verify which regions now constitute the principal centers of production of this commodity, and to examine the public and private actions that support its evolving value chain. The present study thus seeks to respond to the following questions: how has the configuration of coffee production in Brazil changed since the reduction in government intervention in the coffee market? What changes occurred in coffee production between 1984/85 and 2014/15? Which regions are currently specialized in coffee production? Which regions have seen declines in coffee-specialization? And which coffee varieties predominate in each of these regions?

In sum, this study has as its objective the analysis of the dynamics of coffee production in Brazil, including identification of the Brazilian micro-regions specialized in coffee activities, verification of the evolving spatial distribution of productive activities, and evaluation of the economic characteristics of the principal coffee-producing regions.

## Material and methods

In order to analyze the evolution of coffee production across the subset of Brazil’s micro-regions specialized in coffee production, data are drawn from the Municipal Agricultural Survey (PAM), published by the IBGE [1]. Since coffee production occurs over the course of biennia, each two-year grouping (low year and high year) is averaged for all calculations in order to minimize potential variation. Through this method, averages from 1984/85, 1994/95, 2004/05, and 2014/15 are generated.

Initially, Pearson’s correlation coefficient–pair of variables–was applied to analyze the correlation among coffee production in Brazil (t), planted area (hectare), productivity (t/hectare) [1] and the international prices (US$) [9] for the period 1984 to 2016. The formula (Eq 1) is represented as follows:

$$\rho_{XY}=\frac{Cov(X,Y)}{\sigma_{X}\sigma_{Y}}$$ (1)

Where *Cov* denotes the covariance and; *σ*_*X*_ and *σ*_*Y*_ are the standard deviations.

The bivariate Granger-causality tests were applied to determine the causal direction among coffee production in Brazil, planted area, productivity [1] and the international prices [9] for the same period of the Person’s analysis. Causality, as defined by Granger [10], is inferred when lagged values of an independent variable, *X*_*t*_, have the power to explain the regression of a dependent variable, *Y*_*t*_, on lagged values of *Y*_*t*_ and *X*_*t*_.

$$Y_{t}=\sum_{j=-\infty }^{\infty }a_{j}Y_{t-j}+\sum_{j=-\infty }^{\infty }b_{j}X_{t-j}+u_{i}$$ (2)

In the equation above (Eq 2), *b*_*j*_ = 0 for all *j*<0 if and only if *Y*_*t*_ fails to Granger-cause *X*_*t*_. In this paper the test was performed using *t*−1 time lag. The null hypothesis is that *X* does not Granger-cause *Y*. The model was estimated by OLS–Ordinary Lest Square–and the significance was tested using χ^2^−*test*.

The location quotient (LQ) is a measure of relative regional specialization, which is computed from a basic aggregate in order to compare determined activities across locations [11]. For the purposes of this study, this parameter is employed to evaluate specialization in coffee production in Brazilian micro-regions, based on average gross value of production (GVP) for each biennial period included in the analysis. The LQ is computed as the ratio of GVP from coffee over GVP from agriculture, according to Eq 3 [12]:

$$LQ=\frac{\frac{E_{j}^{i}}{E_{j}}}{\frac{E^{i}}{E}}$$ (3)

Where $E_{j}^{i}$ is the GVP of coffee in micro-region *i*; *E*_*j*_ is the total GVP of coffee in country *j*; *E*^*i*^ is the total GVP of agriculture in the micro-region; E is the total GVP of agriculture in the country.

In other words, the numerator represents the participation of GVP of coffee in a determined micro-region as a proportion of the total GVP of coffee in Brazil. Similarly, the denominator represents the participation of total agricultural GVP in the micro-region as a proportion of the total GVP of agriculture in Brazil. Thus, if the result of the LQ calculation is greater than one (LQ ≥ 1), the micro-region is specialized in coffee production, whereas if the LQ is less than one (QL < 1), the micro-region is not specialized in coffee production.

As a further step, Brazilian micro-regions specialized in coffee production during the 2014/15 biennial are subdivided according to the predominant variety produced, arabica or conilon (robusta). This distinction is possible from the year 2012 onwards, when the IBGE began to publish information on variety.

Once Brazilian micro-regions are categorized according to specialization in coffee production, principal-component analysis (PCA) is applied for the 2014/15 biennial. According to [13], PCA measures total variation in the data and finds a linear combination of observed variables that maximally explains the variation. If determined observables are highly correlated, they will be combined into a factor, or component, that explains the largest possible quantity of variation in the sample. The second component will capture the second largest quantity of variation and will not be correlated with the first factor, and so on. To conduct the PCA procedure, data are drawn from the PAM by IBGE and from the Central Bank of Brazil. The variables selected for the PCA were those that have traditionally been used in studies that deal with the dynamics, specialization and spatialization of agricultural production [14]. Variables included in the PCA are: (i) tons of coffee produced; (ii) GVP of coffee in thousands of Brazilian reals; (iii) concentration of area planted with coffee as a proportion of total agricultural area in the micro-region; (iv) LQ of coffee; (v) credit obtained from the National Program for the Strengthening of Family Agriculture (PRONAF) for coffee production; (vi) credit obtained in collaboration with the Coffee Economy Defense Fund (FUNCAFÉ).

PRONAF is a program implemented by the Brazilian Federal Government to stimulate the use of family labor, encouraging the activities of small farmers through a commercial credit line with low interest rates [15]. FUNCAFÉ–regulated by law decree no. 94,874 of September 15, 1987 –is a fund managed by the Brazilian Ministry of Agriculture, Livestock and Supply (MAPA), destined to farmers, cooperatives and agroindustries of the coffee sector, with the purpose of financing the costing, storage, acquisition of coffee and working capital [16].

Based on PCA, cluster analysis of micro-regions is undertaken based on Ward’s Method, which considers the degree of similarity between units. Cluster analysis is a statistical method focused on interdependence, which allows for the grouping of variables into homogenous clusters based on defined parameters and according to a defined measure of similarity or distance. In sum, cluster analysis reduces and clusters principal variables into homogenous groups [17].

All monetary values are in December, 2017 US dollars. Data processing and statistical analyses were conducted using the SPSS 21 software package. The elaboration of maps was undertaken using the software package ArcGIS 10.2.

## Results and discussion

**Fig 1:**
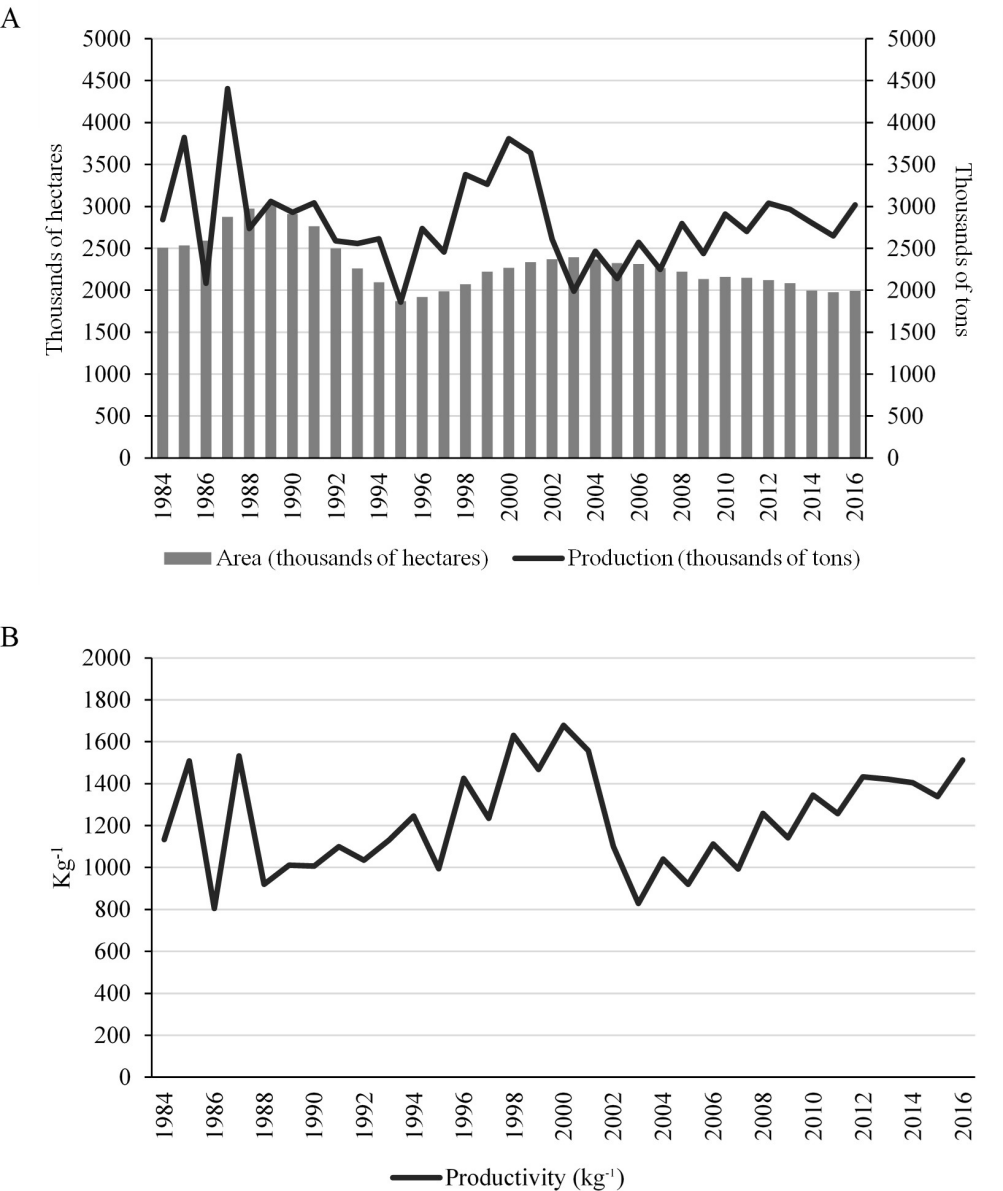
Evolution of area harvested, production, and average productivity for coffee in Brazil over the 1984–2016 period. Source: Created based on data from the IBGE.

Fig 1 illustrates the evolution of harvested area, production volume, and average productivity of coffee in Brazil over the 1984–2016 period. Based on these data, it is evident that, in 1984, harvested coffee area in Brazil was 2.51 million hectares, while in 2016 this value was just 1.99 million hectares, indicating a decline in area of 20.3% over this period. Nonetheless, in terms of production, 2.8 million tons of coffee were produced in 1984, while 3 million tons were produced in 2016, an increase of 6% over the period. This divergence indicates an increase in average land productivity of 33.4% between 1984 and 2016. These productivity gains are the result of technological improvements adopted by producers since 1984, increases in plants per hectare, increased use of agricultural machinery, the development of new varieties [18], and the adoption of irrigation techniques [19]. It is noteworthy that coffee exhibits seasonal variation, with years of high production followed by years of low production and vice versa. This cyclical behavior is related to climatic factors as well as to intrinsic characteristics of the product, which is a perennial [20].

**Fig 2:**
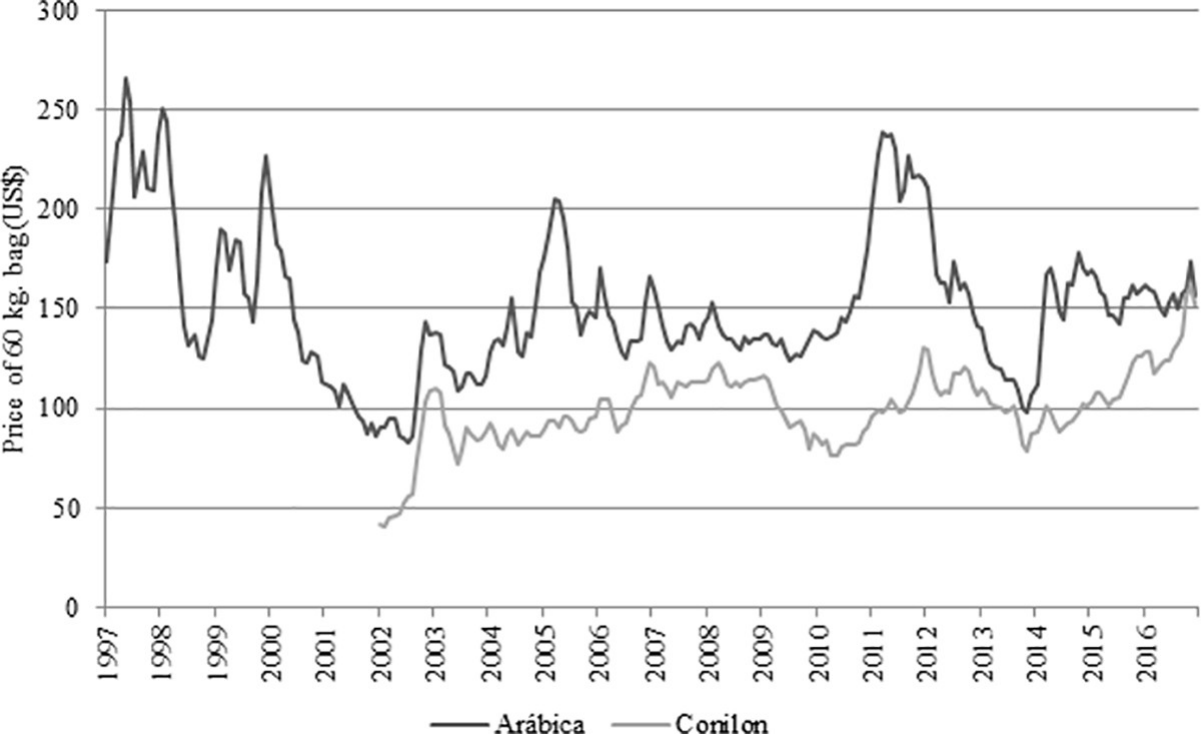
Evolution of price of 60 kg. bag (US$) for arabica and conilon coffee in Brazil over the 2004–2016 period. Source: Created based on data from the Center for Advanced Studies in Applied Economics (CEPEA) of the University of São Paulo (USP). Note: Values were corrected by IPC-A to current values of December, 2017.

It appears that the years 1995 to 2000 encompass the period during which coffee production exhibited its greatest growth, with increases during this period of approximately 104% in production and 21.3% in harvested area, after which this latter variable then proceeds to fall sharply until 2003. Since coffee prices per bag were low in the immediate post-2000 period (Fig 2), coffee producers did not feel encouraged to adequately manage their coffee plantations, which consequently resulted in a 39.12% fall in production. In 1999, a bag of arabica coffee cost on average US$ 144.43 on the Brazilian market, while in 2002 a bag of the same variety cost US$85.45. The low price may be explained by the entrance into the market of new international producers, and by the adoption of misguided policies, culminating in inflated stockpiles of coffee in consumer countries. Furthermore, with the dismantling of the IBC, Brazil became exposed to the influence of futures markets in the determination of domestic coffee prices, which on one hand offered greater security to agents involved in the commercialization of coffee, and on the other hand, it resulted in coffee price instability that generated substantial insecurity among producers [21].

In general, conilon (robusta) coffee exhibited lower prices compared to arabica coffee [22]. Nonetheless, between 2004 and 2016, both arabica and conilon varieties exhibited growth tendencies. In 2016, as a result of a fall in conilon production due to a drought in the state of Espírito Santo (conilon production levels were 27.7% lower in 2016 than in 2015), the price of conilon coffee reached prices nearly as high as those of arabica. Increasingly, inclusion of conilon beans in gourmet coffee blends has increased their exposure in Brazil, as well as in exports [23].

At this point in the paper, we are interested in how the variables production of coffee in Brazil (t), planted area (hectare), productivity (t/hectare) and the international price (US$) are related. Pearsons’s correlation coefficient (Table 1) indicates statistically significant correlation between some of these variables–for the entire country overtime (1984–2016).

**Table 1:** Pearson’s correlation coefficients matrix.

	Production	Planted area	Productivity	Price
Production	1			
Planted area	0.29**	1		
Productivity	0.78*	-0.35*	1	
Price	-0.11	-0.36*	0.13	1

Source: Created based on data from the IBGE and International Monetary Fund. Note

* significance level *p*<0.05.

** significance level *p*<0.10.

The correlation between production and planted area (0.29), production and productivity (0.78), planted area and productivity (-0.35) and planted area and prices (-0.36) are statistically significant, indicating that variables production, planted area, production and prices are correlated. To reinforce the correlation analysis, the Granger-causality test (Table 2) explores the direction of the causality.

**Table 2:** Granger causality test.

Dependent variable	Independent variable	*χ*^2^	*p*−*value*
Production	Productivity	0.02	0.86
Productivity	Production	10.60*	0.00
Production	Planted area	0.26	0.60
Planted area	Production	0.84	0.35
Production	Price	6.50**	0.010
Price	Production	0.16	0.68
Productivity	Planted area	8.2*	0.00
Planted area	Productivity	0.88	0.34
Productivity	Price	11.56*	0.00
Price	Productivity	1.33	0.24
Planted area	Price	5.23*	0.02
Price	Planted area	2.7**	0.10

Source: Created based on data from the IBGE and International Monetary Fund. Note

* significance level *p*<0.05.

** significance level *p*<0.10.

The findings suggest that coffee production Granger-causes coffee productivity–economies of scale–, and prices Granger-causes production, productivity and planted area, which is plausible analyzing Brazilian coffee market (Table 2). Therefore, the key element of the Brazilian coffee agrichain is the price and the bases of the Brazilian coffee competitiveness are the economies of scale, resulting, in a context of a deregulated and with high level of competition market, low prices. Therefore, specialized area is a kind of strategy to gain competitiveness and economies of scale as a response for prices. In an unregulated market with high level of competition the strategies of quality and differentiation have played a pivotal role in aggregating value.

Brazilian coffee production has experienced important changes driven by new consumer markets that are increasingly focused on production processes and product quality. According to [24], there is strong growth in segments of the consumer coffee market that demand greater transparency and information for the consumer, which in turn has allowed geographical certification to add increasing value to final products. Likewise, coffee that is sold without accompanying geographic labeling tends to be considered of inferior quality in the market, while products that offer greater information are considered to be of higher quality. In response to these changing preferences, organizations such as the Brazilian Specialty Coffee Association (BSCA), and programs such as the Seal of Purity and Quality Program (PQC), the Sustainable Coffees of Brazil Program, and the Common Code for the Coffee Community (4C) support producers’ attempts to obtain certifications and recognitions for their products [25].

**Fig 3:**
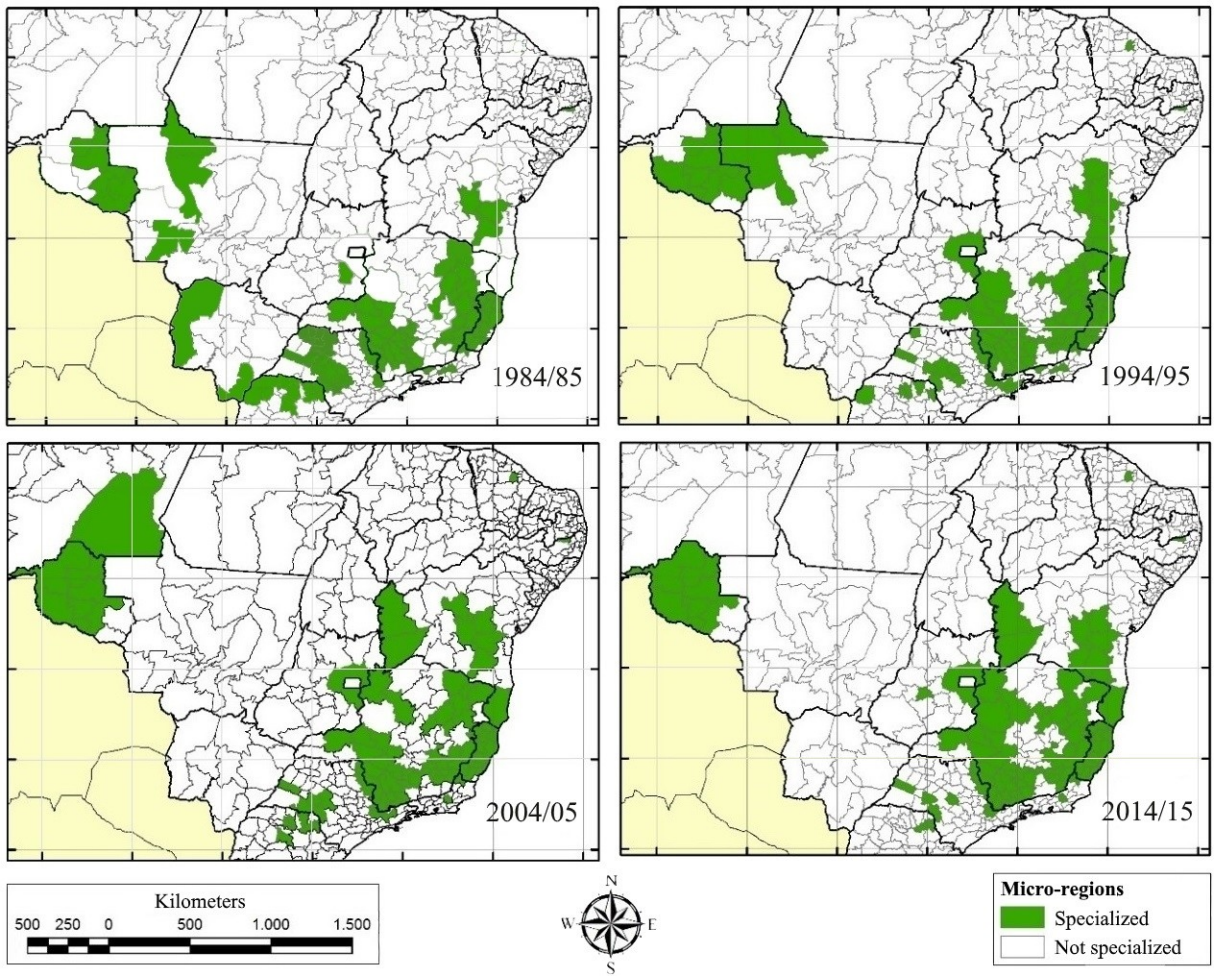
Brazilian micro-regions specialized in coffee production, in the 1984/85, 1994/95, 2004/05, and 2014/15 biennia. Source: Created based on data from the IBGE.

Based on the identification of micro-regions specialized in coffee production, it is possible to observe their respective levels of development, and thereby verify which still require more resources and technical support in order to improve their production and guarantee their ability to supply the market with quality, valuable products. These factors are important for the broader development and sustainability of the coffee production chain in Brazil. Fig 3 presents the Brazilian micro-regions specialized in coffee production during the 1984/85, 1994/95, 2004/05, and 2014/15 biennia. During the 1984/85 period, there were 102 micro-regions specialized in coffee production, for 1994/95 there was a slight decline to 94, while in 2004/05 there was a slight decline to 92, and a further slight decline to 90 by 2014/15.

Although there has not been an increase in the number of specialized micro-regions in Brazil over this period, there have been important transformations in the spatial distribution of coffee production in the country. The states of Paraná and São Paulo, which in the past were the major national producers of this commodity [26] [27], collectively accounted for only 12.12% of national production during the 2014/15 biennium. Specialized micro-regions have increasingly become concentrated in four states: Minas Gerais, Espírito Santo, Bahia, and Rondônia. Together, these four states hosted approximately 80% of the specialized micro-regions, and represented 82% of total production in the country in 2014/15. In other words, the primary producing states were those that concentrated the greatest number of specialized micro-regions.

**Fig 4:**
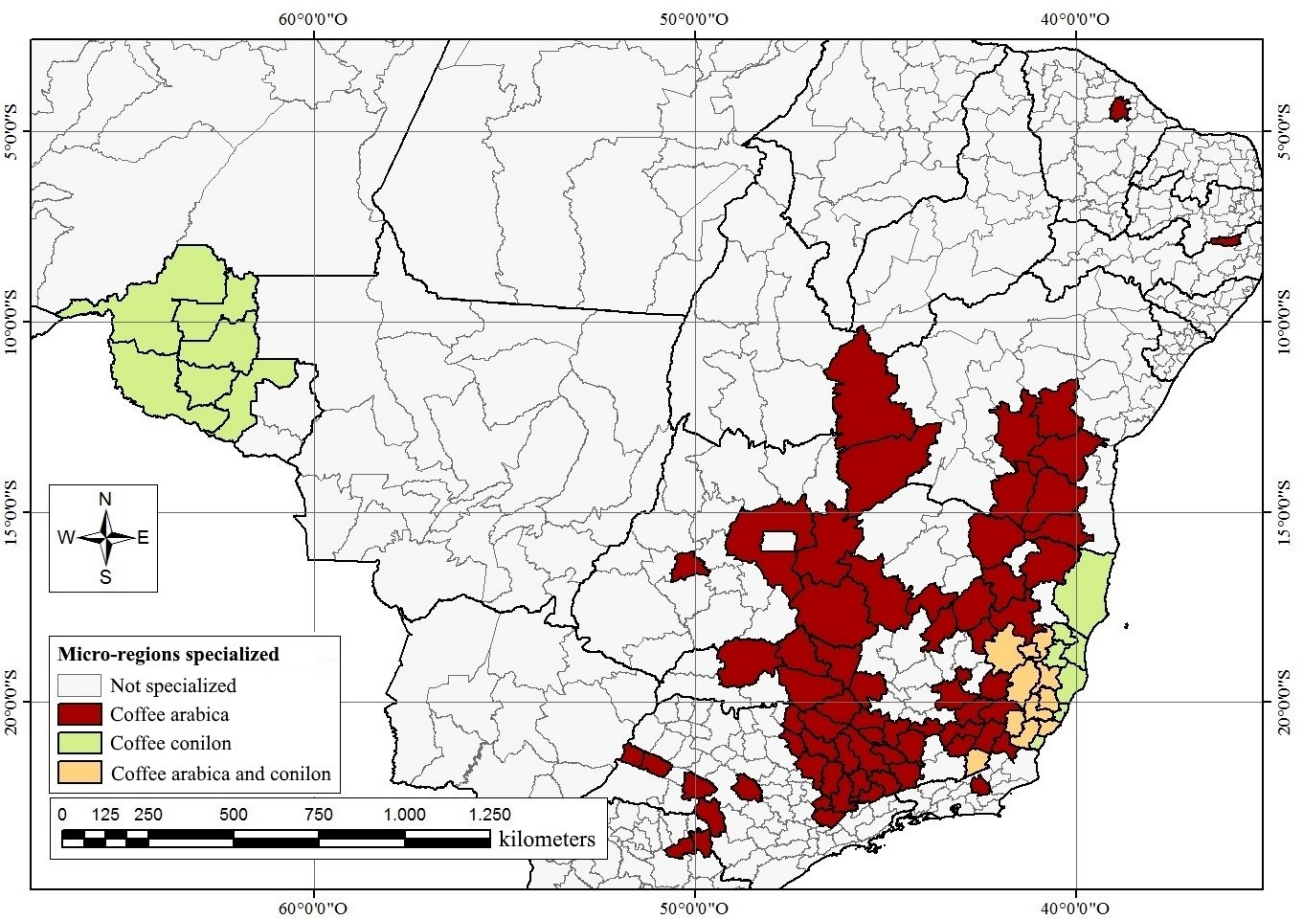
Distribution of arabica and conilon coffee varieties throughout the specialized coffee-producing micro-regions of Brazil, 2014/15. Source: Created based on data from the IBGE.

Fig 4 illustrates the Brazilian micro-regions specialized in coffee production by variety–arabica, conilon, or both, in the 2014/15 biennium. Of all the micro-regions specialized in coffee production, 66 specialized solely in arabica, 13 solely in conilon, and 11 in both.

The micro-regions specialized in arabica production are predominantly concentrated in the *Cerrado* region of the Southeast and the state of Bahia. The state of Minas Gerais exhibited the largest number of micro-regions specialized in arabica (60%), and arabica coffee accounted for 75.4% of total coffee production, and 81.4% of GVP from coffee production in this state. These numbers highlight the importance of Minas Gerais and the *Cerrado* region within the coffee economy of Brazil. These regions host modern, high-productivity coffee production, favorable landscapes, and extensive use of irrigation and mechanization [19].

Micro-regions specialized in conilon coffee are primarily concentrated in the states of Espírito Santo and Rondônia. The state of Espírito Santo is the largest producer of this species, accounting for 54.2% of all conilon-specialized micro-regions in Brazil. According to [28], Espírito Santo stands out for its ideal temperature conditions–nearly the entire territory of the state is amenable to conilon coffee cultivation.

In the 2014/15 biennium, conilon cultivation was present in all of the micro-regions of Espírito Santo, and occupied an area of approximately 290,000 hectares, which constitutes 65% of all area dedicated to coffee production in the state. A further 25% of micro-regions specializing in conilon production were located in the state of Rondônia in 2014/15. The conilon coffee cultivation in low-altitude, high-temperature areas, such as Rondônia, has expanded rapidly in recent decades, and served as the primary source of income for 38,000 small farming households in 2014/15 [29]. These numbers reveal the important role played by coffee production in Rondônia’s rural economy.

Table 3 presents coefficients of linear combinations from PCA. Two principle components were identified through PCA, which together explain 73.08% of the variation in the data. Component 1 explains 37.56% of variation in the data and is positively correlated with the following variables: production of arabica coffee, GVP of arabica coffee, and concentration of area planted with arabica coffee. This component may therefore be called the “arabica component.” Component 2 explains 35.53% of the variation in the data, and is positively correlated with the following variables: LQ, production of conilon coffee, GVP of conilon coffee, concentration of area planted with conilon coffee, PRONAF, and FUNCAFÉ. In other words, Component 2 (which may be called the “conilon component”) is composed of micro-regions specialized in conilon coffee production and sources of rural credit.

**Table 3:** Principal-component analysis–coefficients of linear combinations.

Variable	Component	
	1	2
LQ	0,454	0,730
Production of conilon coffee	-0,518	0,723
Production of arabica coffee	0,811	0,267
GVP of conilon coffee	-0,577	0,665
GVP of arabica coffee	0,848	-0,151
Concentration areas of conilon coffee	-0,530	0,788
Concentration areas of arabica coffee	0,782	0,305
PRONAF	0,353	0,675
FUNCAFÉ	0,423	0,661

Notes: LQ: Location Quotient. GVP: Gross Value of Production. PRONAF: *Programa Nacional de Fortalecimento da Agricultura Familiar*. PRONAMP: *Programa Nacional de Apoio ao Médio Produtor Rural*.

**Fig 5:**
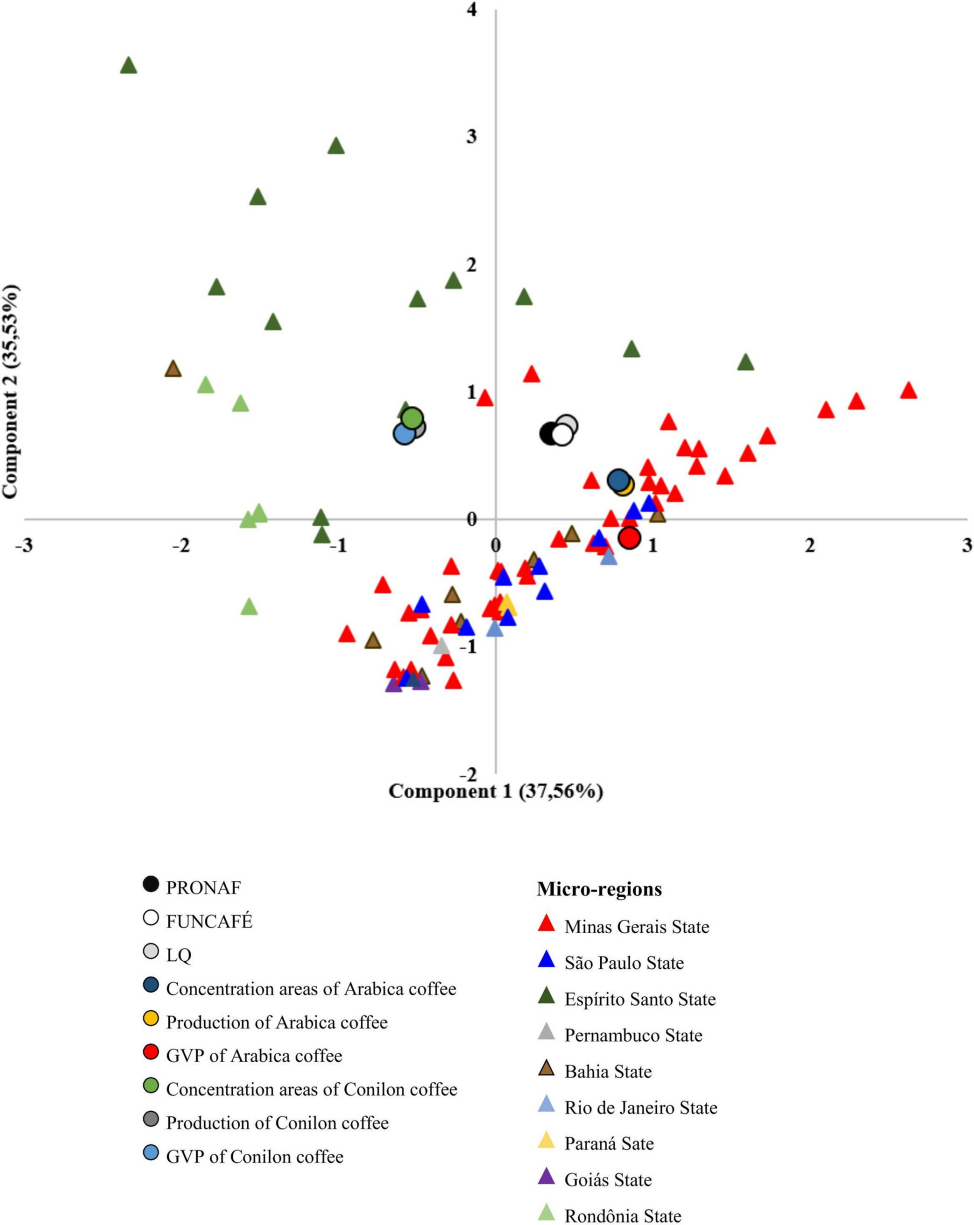
Principal-component analysis. Source: Created based on data from the IBGE and Central Bank of Brazil.

Fig 5 presents results from PCA along with micro-regions specialized in coffee production for the 2014/15 biennium. From the graph, it is apparent that micro-regions specialized in arabica coffee production (exhibiting greater productivity, production, GVP, and concentration of area dedicated to arabica) are correlated with Component 1, while micro-regions specialized in conilon production (exhibiting greater productivity, production, GVP, and concentration of area dedicated to conilon) are correlated with Component 2.

The majority of the micro-regions in Minas Gerais, as well as some in São Paulo and Espírito Santo, are strongly associated with variables related to arabica (concentration of area, production, and GVP), while the micro-regions of Espírito Santo and Rondônia are more strongly associated with variables related to conilon (concentration of area, production, and GVP). Furthermore, conilon also exhibits strong correlation with variables measuring rural credit: PRONAF and FUNCAFÉ.

**Fig 6:**
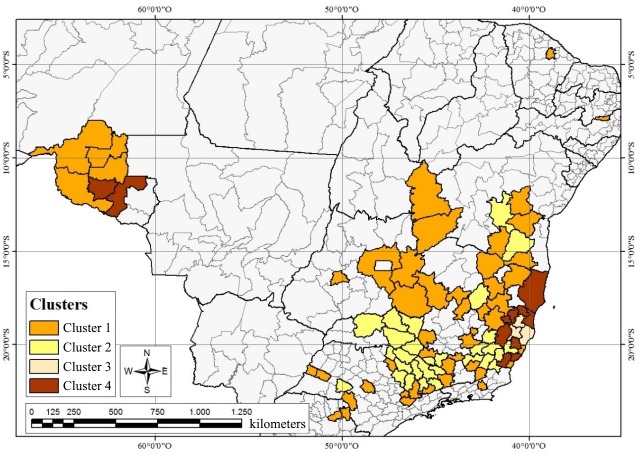
Map of clusters of Brazilian micro-regions specializing in coffee production.

Fig 6 presents a map of clusters of specialized coffee-producing micro-regions in Brazil. Based on degree of similarity across covariates, four clusters were identified. Group 1 is composed of 47 micro-regions, Group 2 of 29 micro-regions, Group 3 of 3 micro-regions, and Group 4 of 11 micro-regions.

Group 1 is composed of 47 micro-regions distributed throughout the states of Bahia (6), Ceará (1), Espírito Santo (2), Goiás (2), Minas Gerais (21), Pernambuco (1), Paraná (2), Rio de Janeiro (1), Rondônia (4), and São Paulo (7). This group is dominated by micro-regions specialized in arabica production, which exhibit low use of rural credit programs, indicating the predominance of commercial agriculture. The majority of the micro-regions in Group 1 are located in the Brazilian *Cerrado* and part of the state of Rondônia (the Madeira-Guaporé meso-region). The *Cerrado* region of Minas Gerais has become one of the most advanced and productive coffee-producing regions in the country, exhibiting extensive adoption of innovative technologies [30], that is, with intensive use of agricultural tools and modern management techniques [31]. In relation to coffee production in Rondônia, it has consistently been the state’s most important perennial crop (in terms of cultivated area) since early settlement of the region [29].

Group 2 is composed of 29 micro-regions distributed across the states of Bahia (2), Espírito Santo (2), Minas Gerais (21), Rio de Janeiro (1), and São Paulo (3). Group 2 is also formed by micro-regions specialized in arabica production, but in contrast to Group 1, Group 2 is characterized by high levels of access to rural credit programs. The group includes diverse micro-regions from the south of Minas Gerais and the Mogiana Paulista region. These regions are among the best in the world in terms of product quality, which may be attributed to a favorable climate and modern techniques of cultivation and administration, which enable the harvest of a refined product favorably differentiated from that produced in other regions of the country [32]. In the south of Minas Gerais, the main region of the group, there is the predominance of mechanization in flat areas and low use of machinery in the mountains, in addition, there is a mix of mechanization and hired labor, but still with low qualifications [31].

Group 3 consists of only 3 micro-regions from the state of Espírito Santo (Linhares, Colatina, and Nova Venécia). These regions specialize in conilon cultivation and exhibit high levels of production and use of rural credit programs, indicating the predominance of family agriculture. Espírito Santo faces limits to productivity resulting from a hydrological deficit, but nonetheless enjoys ideal temperatures for conilon cultivation, making this state the largest producer of conilon in Brazil and accentuating the importance of its specialized coffee-producing micro-regions [28].

Group 4 is composed of micro-regions spread across the states of Bahia (1), Espírito Santo (6), Minas Gerais (2), and Rondônia (2). Micro-regions in this group are specialized in conilon production, characterized by demand for rural credit, and primarily located in the Vale do Rio Doce and Vale do Mucuri meso-regions of Minas Gerais and the state of Espírito Santo. The state of Espírito Santo offers ideal conditions for coffee cultivation, who highlights the potential of coffee regions in Northern Minas Gerais, including the Vale do Jequitinhonha and Vale do Mucuri, to produce high quality coffee [28].

Based on the composition of these clusters, it is evident that the state of Minas Gerais is the principal coffee-producing area in Brazil. Analysis of the dynamics of coffee production in the country reveals that there is substantial heterogeneity among coffee-producing regions, be it in terms of variety cultivated, system of production, or use of rural credit. Even within the state of Minas Gerais, substantial diversity in technological advancement and farm ownership structure persists. More broadly, the coffee production value chain has played a central role in the development of Minas Gerais, generating positive externalities and spillovers into other sectors of the economy [33].

Analysis of Brazil’s agricultural development over the course of the 20^th^ and 21^st^ Centuries reveals that the country transitioned from traditional agriculture, characterized by low capital-intensity, to more highly-developed, technology intensive production [30]. This broader process of development has been driven by transformations along diverse agricultural production chains, including that of coffee. Brazilian coffee production, while heterogeneous, has achieved increases in quality even while adapting to structural transformations resulting from the dismantling of government intervention in the sector. Coffee producers are increasingly focused on satisfying evolving consumer preferences, seeking certifications, geographical differentiation, and quality seals for their products. Together, these elements express the constantly evolving dynamics of coffee production in Brazil.

## Conclusions

Brazil did not experience substantial change in the number of micro-regions specialized in coffee production over the 1984–2015 period. Nonetheless, the country did undergo important changes in the spatial distribution of production. While the traditional coffee-growing states of Paraná and São Paulo maintained only a few specialized coffee-producing regions, the states of Minas Gerais and Espírito Santo experienced dramatic growth, transforming these states into the most important coffee producers in the country. The states of Bahia and Rondônia also emerged as important contributors to national coffee production, and helped to further solidify the central role of coffee in the Brazilian agro-economy.

Micro-regions specializing in the production of arabica varieties are concentrated in the states of Minas Gerais and Bahia, while the production of conilon (robusta) coffee predominates in Rondônia. Espírito Santo hosts micro-regions specializing in both arabica and conilon production. Evidently, Brazilian coffee production is characterized by a high degree of heterogeneity.

Based on Principal-Component Analysis, two principal components were identified which, together, explain 73.08% of the variation in the data. Component 1 explains 37.56% of the variation in the data and is positively correlated with variables related to arabica coffee. In contrast, Component 2 explains 35.53% of the variation in the data and is positively correlated with variables related to conilon coffee and rural credit.

Cluster analysis revealed four groups distinguished by divergent systems of production, variety cultivated, use of rural credit, and degree of specialization in coffee production. Group 1 was composed of micro-regions specialized in arabica production, with a predominance of commercial producers. Group 2 was also composed of micro-regions specialized in arabica production, but with a predominance of family producers. Group 3 contained micro-regions specializing in conilon production and featuring primarily family producers making extensive use of rural credit, while Group 4 was composed of micro-regions specialized in conilon production, with family producers making less use of rural credit.

The main effects of the international coffee market deregulation, occurred in the early 1990’s, were the increase in the price volatility and the decrease in the prices levels and producers income in this agrichain. Thus, the strategies of governance of the value chain were very important, and in the case of Brazil the low cost of production and economies of scale were the main element of competitiveness. As a whole, the dynamics of coffee production in Brazil have included significant shifts in the spatial distribution of specialized coffee-producing micro-regions as a response to an unregulated market and a new price dynamics post-1990, as well as evolution and variation in coffee production systems and species. These transformations have emerged from a context of structural transformation of the Brazilian coffee sector and evolution in the economic importance of each micro-region specialized in coffee activities.
